# Effect of a tumour-produced lipid-mobilizing factor on protein synthesis and degradation

**DOI:** 10.1054/bjoc.2001.1834

**Published:** 2001-06

**Authors:** B S Islam-Ali, M J Tisdale

**Affiliations:** Pharmaceutical Sciences Research Institute, Aston University, Birmingham B4 7ET, UK

**Keywords:** lipid mobilizing factor, protein synthesis, cyclic AMP, β-adrenergic receptor

## Abstract

Treatment of murine myoblasts, myotubes and tumour cells with a tumour-produced lipid mobilizing factor (LMF), caused a concentration-dependent stimulation of protein synthesis, within a 24 h period. There was no effect on cell number or [^3^H] thymidine incorporation, but a similar concentration-dependent stimulation of 2-deoxyglucose uptake. LMF produced an increase in intracellular cyclic AMP levels, which was linearly (r^2^= 0.973) related to the increase in protein synthesis. The effect of LMF was attenuated by the adenylate cyclase inhibitor MDL _12330A_, and was additive with the stimulation produced by forskolin. Both propranolol (10 μM) and the specific β_3_-adrenergic receptor antagonist SR 59230A (10^–5^M), significantly reduced the stimulation of protein synthesis induced by LMF. Protein synthesis was also increased by 69% (*P* = 0.006) in soleus muscles of mice administered LMF, while there was a 26% decrease in protein degradation (*P* = 0.03). While LMF had no effect on the lysosomal enzymes, cathepsins B and L, there was a decrease in proteasome activity, as determined both by the ‘chymotrypsin-like’ enzyme activity, as well as expression of proteasome α-type subunits, determined by Western blotting. These results show that in addition to its lipid-mobilizing activity LMF also increases protein accumulation in skeletal muscle both by an increase in protein synthesis and a decrease in protein catabolism. © 2001 Cancer Research Campaign http://www.bjcancer.com


*British Journal of Cancer* (2001) **84**(12), 1648–1655
© 2001 Cancer Research Campaign
doi: 10.1054/ bjoc.2001.1834, available online at http://www.idealibrary.com on IDEAL®

### Effect of a tumour-produced lipid-mobilizing factor on protein synthesis and degradation

**BS Islam-Ali and MJ Tisdale**

Pharmaceutical Sciences Research Institute, Aston University, Birmingham B4 7ET, UK

**Summary** Treatment of murine myoblasts, myotubes and tumour cells with a tumour-produced lipid mobilizing factor (LMF), caused a concentration-dependent stimulation of protein synthesis, within a 24 h period. There was no effect on cell number or [$^3$H] thymidine incorporation, but a similar concentration-dependent stimulation of 2-deoxyglucose uptake. LMF produced an increase in intracellular cyclic AMP levels, which was linearly ($r^2$ = 0.973) related to the increase in protein synthesis. The effect of LMF was attenuated by the adenylate cyclase inhibitor $MDL_{12330A}$, and was additive with the stimulation produced by forskolin. Both propranolol (10 μM) and the specific $\beta_3$-adrenergic receptor antagonist SR 59230A ($10^{-5}$M), significantly reduced the stimulation of protein synthesis induced by LMF. Protein synthesis was also increased by 69% ($P$ = 0.006) in soleus muscles of mice administered LMF, while there was a 26% decrease in protein degradation ($P$ = 0.03). While LMF had no effect on the lysosomal enzymes, cathepsins B and L, there was a decrease in proteasome activity, as determined both by the 'chymotrypsin-like' enzyme activity, as well as expression of proteasome α-type subunits, determined by Western blotting. These results show that in addition to its lipid-mobilizing activity LMF also increases protein accumulation in skeletal muscle both by an increase in protein synthesis and a decrease in protein catabolism. © 2001 Cancer Research Campaign http://www.bjcancer.com

**Keywords**: lipid mobilizing factor; protein synthesis; cyclic AMP; β-adrenergic receptor

Cancer cachexia is associated with the progressive depletion of skeletal muscle mass and adipose fat deposits. We have recently isolated 2 catabolic factors produced by cachexia-inducing tumours that have the potential for inducing these changes in body composition: (i) a proteolysis-inducing factor (PIF), which acts on skeletal muscle to induce protein degradation and to inhibit protein synthesis (Lorite et al, 1997). (ii) a lipid-mobilizing factor (LMF), which acts directly to stimulate triglyceride hydrolysis in adipocytes by stimulation of adenylate cyclase in a GTP-dependent process (Hirai et al, 1998). Administration of purified PIF to mice produces weight loss with depletion of skeletal muscle without an effect on adipose tissue (Todorov et al, 1996), while LMF produces a specific reduction in carcass lipid with a tendency to increase the non-fat carcass mass (Hirai et al, 1998). Induction of lipolysis in epididymal adipocytes is attenuated by the β-adrenergic receptor (β-AR) antagonist propranolol (Khan and Tisdale, 1999), suggesting that the action of LMF may be mediated through a β-AR. β-Agonists stimulate skeletal muscle hypertrophy in animals (Mersmann, 1998) and some have been reported to increase protein synthesis in multinucleated muscle cells in culture (Anderson et al, 1990; Grant et al, 1990; McMillan et al, 1992). The mechanism requires an increase in cyclic AMP, although the pathways leading from cyclic AMP to changes in protein synthesis are incompletely understood. However, phosphorylation of 40S ribosomal protein S6 is elicited in a wide variety of cells and correlates with increased rates of translation (Stefanovic et al, 1986).

*Received 10 January 2001*
*Revised 13 March 2001*
*Accepted 15 March 2001*

*Correspondence to:* MJ Tisdale

This study examines the effect of LMF on protein synthesis and degradation in $C_2C_{12}$ murine myoblasts and in soleus muscle of mice, and attempts to elucidate the role of cyclic AMP in this process.

### MATERIALS AND METHODS

### Chemicals and radiochemicals

L-[2, 6-$^3$H] Phenylalanine (sp. act. 2.07 TBq mmol$^{-1}$), 2-deoxy-D [2, 6-$^3$H] glucose (sp. act. 1.63 TBq mmol$^{-1}$) and 8[$^3$H] cyclic AMP (sp. act. 888 GBq mmol$^{-1}$) were purchased from Amersham Lifesciences, Bucks, UK. 5-Methyl [$^3$H] thymidine (sp. act. 3.1 TBq mmol$^{-1}$) was purchased from NEN Research Products, Herts, UK. $MDL_{12330A}$, H8, rapamycin, PD 98059, wortmannin, LY 294002 and forskolin were purchased from Calbiochem, Nottingham, UK, while cyclic AMP-dependent protein kinase was purchased from Sigma Chemical Co, Dorset, UK. SR 59230A was a gift from Dr L Manara, Sanofi Winthrop, Italy. Fetal calf serum (FCS) and Dulbecco's modified Eagle's medium (DMEM) were purchased from Life Technologies, Paisley, Scotland.

### Cell culture

$C_2C_{12}$ murine myoblasts were cultured in DMEM supplemented with 12% FCS, 1% penicillin-streptomycin, in a humidified atmosphere of 5% $CO_2$ in air at 37˚C. MAC16 cells were maintained in RPMI 1640 medium containing 5% FCS. All experiments were performed with the cells in a sub-confluent state.

### Purification of LMF

LMF was purified from the urine of cachectic patients with pancreatic carcinoma using a combination of batch extraction on

DEAE-cellulose and hydrophobic interaction chromatography (Todorov et al, 1996 ) Particulate material was removed from urine by centrifugation at 3000 ***g*** for 10 min followed by dilution with 4 vol 10 mM Tris. HCl, pH 8.0. DEAE cellulose (10 g $l^{-1}$ of original urine) was added and the mixture was stirred for 2 h at 4˚C. The DEAE-cellulose was recovered by low speed centrifugation and LMF bioactivity, determined by glycerol release from epididymal adipocytes (Khan and Tisdale, 1999), was eluted with 0.5 M NaCl in 10 mM Tris. HCl, pH 8.0. The eluate was equilibrated against PBS and concentrated to 1 ml before further purification using a Resource-Iso HPLC column (Pharmacia Biotech, St Albans, Herts, UK) employing a decreasing $(NH_4)_2SO_4$ concentration from 1.5 M. Active fractions containing LMF eluted at 0.6 M $(NH_4)_2SO_4$, and were desalted before use by washing 5 times against PBS using an Amicon filtration cell. LMF eluted as a single protein band of $M_r$ 43000 as determined by Coomassie blue staining of a 12% SDS polyacrylamide gel (Figure 1).

### Precursor incorporation

$C_2C_{12}$ myoblasts were seeded at $5 \times 10^4$ cells $ml^{-1}$ and MAC16 at $10^5$ cells $ml^{-1}$ in 6-well multidishes containing 2 ml medium per well. After 24 h various concentrations of LMF was added and left for a further 24 h period. During the last 60 min of incubation the cells were incubated with 1.5 µmol L-phenylalanine containing 37 kBq of L-[2, 6-$^3$H] phenylalanine. The reaction was terminated by removal of medium and washing the cells 3 times with ice-cold PBS. The cells were then incubated at 40˚C for 20 min with 1 ml ice-cold 0.2 M perchloric acid, which was replaced with 1 ml 0.3 M NaOH and incubation continued at 4˚C for a further 30 min, followed by a further 20 min at 37˚C. Cellular protein was precipitated with 2 M perchloric acid (0.5 ml) for 20 min at 4˚C, followed by centrifugation at 3000 ***g*** for 10 min at 4˚C. The pellet, which comprised DNA and protein was dissolved in 1 ml of 0.3 M NaOH, and an aliquot (20 µl) was used to measure protein concentration using the Bio-Rad reagent (Sigma Chemical Co, Dorset, UK). The radioactivity in 0.5 ml was determined using a 2000CA Tri-Carb liquid scintillation analyser. The rate of protein synthesis was calculated as dpm µg $protein^{-1}$ $h^{-1}$ as described (Southorn and Palmer, 1990). The protocol for DNA synthesis was the same as above except that methyl [$^3$H] thymidine (0.5 µCi) was added at the same time as LMF and left for 48 h. Protein was precipitated using ice-cold 5% trichloroacetic acid for 1 h at 4˚C. Incorporation of 2-deoxy-D [2, 6-$^3$H] glucose ([$^3$H] 2DG) was determined in $C_2C_{12}$ myoblasts 24 h after addition of LMF. The media was removed, and the cells were rinsed once with Krebs Ringer bicarbonate buffer, followed by incubation for 30 min at 37˚C in a further 1 ml of Krebs Ringer bicarbonate, together with 0.1 mM [$^3$H] 2DG (sp. act 74 MBq $mmol^{-1}$). Cells were washed 3 times with ice-cold PBS and incubated on ice for 1 h with lM NaOH (1 ml) and the amount of radioactivity incorporated was determined.

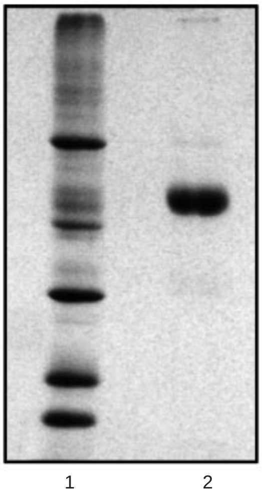

**Figure 1** Electrophoretic separation of proteins on a 12% SDS polyacrylamide gel. Lane 1 molecular weight markers; Lane 2 LMF (5 µg protein) eluted from Resource-Iso column at 0.6 M $(NH_4)_2SO_4$

### Cyclic AMP determination

$C_2C_{12}$ myoblasts were seeded at $4 \times 10^4$ cells $ml^{-1}$ in 1 ml medium in a 24-well multi-dish and left for 48 h before addition of LMF for 30 min at 37˚C. The medium was removed and replaced with 0.5 ml 20 mM HEPES, pH 7.5, 5 mM EDTA and 0.1 mM isobutylmethylxanthine and then the cells were heated on a boiling water bath for 5 min, followed by cooling on ice for 10 min. The cell extracts were sonicated on ice followed by centrifugation at 5000 rpm for 15 min. To 50 µl of the cell extract was added 925 Bq of [8-$^3$H] cyclic AMP and 20 µg of cyclic AMP-dependent protein kinase and incubated for 2 h at 4˚C. Unbound cyclic AMP was removed by adsorption onto charcoal and the concentration of cyclic AMP in the sample determined by comparison with standard curves using known concentrations of cyclic AMP.

### Determination of the activation of protein kinase A (PKA)

$C_2C_{12}$ myoblasts were treated with LMF for 24 h and cytosolic fractions were produced by sonication in 0.25 M sucrose, 1 mM EDTA, 1 mM dithiothreitol, 10 µg $ml^{-1}$ leupeptin and 10 µg $ml^{-1}$ antipain followed by high speed centrifugation. The activation of PKA in the cellular supernatants was determined using the Pierce colourimetric PKA assay kit, Spinzyme™ Format (Rockford, IL, USA) using peptide substrate (Kemptide) labelled with fluorescent dye. The phosphorylated product was quantitated by measuring absorbance at 570 nm.

### Measurement of proteasome activity

$C_2C_{12}$ myoblasts were treated with different concentrations of LMF for 24 h and the activity of the 26S proteasome was determined according to the method of Orino et al (1991). Cellular supernatants were prepared in 20 mM Tris. HCl, pH 7.5, 2 mM ATP, 5 mM $MgCl_2$ and 1 mM dithiothreitol and incubated with the fluorescent substrate succinyl-LLVY-MCA (0.1 mM) in 100 mM Tris. HCl, pH 8.0. The reaction was terminated by the addition of 80 mM sodium acetate and the fluorescence was measured with an excitation of 360 nm and an emission of 460 nm. The protein concentration of the sample was determined using the Bradford assay (Sigma Chemical Co, Dorset, UK).

### Western blot analysis

Samples (5 µg protein) were resolved on 10% sodium dodecylsulfate, polyacrylamide gels and transferred to 0.45 µm nitrocellulose membranes (Hybond A, Amersham, UK), which had been blocked

*© 2001 Cancer Research Campaign*

with 5% Marvel in Tris buffered saline (TBS) at 4˚C overnight. The primary antibodies used were mouse MCP231, a mouse monoclonal antibody to 20S proteasome subunits α1, 2, 3, 5, 6 and 7 (Affiniti Research Products, Exeter, UK), and mouse monoclonal antibody to myosin (Novacastra, Newcastle-upon-Tyne, UK), at a dilution of 1:1000 and 1:200 respectively. The secondary antibody was peroxidase-conjugated rabbit anti-mouse (Dako Ltd, Cambridge, UK) used at a 1:2000 dilution. Incubation was carried out for 1 h at room temperature, and development was by enhanced chemiluminescence (ECL) (Amersham, UK).

### Determination of protein synthesis and protein degradation in soleus muscle after LMF administration

Ex-breeder male NMRI mice (average weight 44.50 g) were administered LMF (8 μg, b.d. iv) for 48 h. After termination soleus muscles were isolated and both protein synthesis and degradation were determined essentially as described (Smith and Tisdale, 1993). Protein synthesis was determined by the incorporation of L-[4-$^3$H] phenylalanine into protein during a 2 h incubation, and the rate of protein synthesis was calculated by dividing the amount of protein bound radioactivity by the amount of acid soluble radioactivity. Protein catabolism was determined by the tyrosine release assay as described (Lorite et al, 1997). Tyrosine release from soleus muscles was determined during a 2 h incubation in Krebs–Henseleit buffer containing 6 mM D-glucose, 1.2 mg ml$^{-1}$ bovine serum albumin and 130 μg ml$^{-1}$ cycloheximide. Tyrosine was quantitated by a fluorometric method (Waalkes and Undenfriend, 1957) at 570 nm on a Perkin–Elmer LS-5 luminescence spectrometer. The animal ethics meet the standards required by the UKCCCR Guidelines.

### Assay of cathepsins L and B

$C_2C_{12}$ myoblasts pre-incubated for 24 h with LMF were homogenized in 250 mM sucrose, 2 mM EGTA, 2 mM EDTA, 20 mM Tris. HCl, pH 7.4, containing 0.2% Triton X-100 followed by sonication. The supernatants formed after centrifugation at 18 000 ***g*** for 15 min were used to determine cathepsin activity as described (Lorite et al, 1998) using the fluorometric substrates N-CBZ-Phe-Arg-7-amids-4-methylcoumarin for cathepsin L and N-CBZ-Arg-Arg-7-amido-4-methylcoumarin for cathepsin B. The flourescence of the free aminomethylcoumarin was determined at an excitation wavelength of 370 nm and an emission wavelength of 430 nm.

### RESULTS

A dose–response curve showing the effect of increasing concentrations of LMF on protein synthesis in $C_2C_{12}$ myoblasts is shown in Figure 2A. Protein synthesis was increased in a concentration-dependent manner, with a maximal 40% stimulation above control values at a concentration of LMF of 580 nM ($P$ < 0.001 from control). A similar dose–response relationship was obtained for LMF on protein synthesis in $C_2C_{12}$ myotubes (Figure 2B), and in MAC16 cells (Figure 2C). LMF also produced a stimulation of 2-deoxyglucose uptake into $C_2C_{12}$ myoblasts (Figure 2D) and MAC16 tumour cells (Figure 2E) with a dose–response curve similar to that for stimulation of protein synthesis. There was no effect of LMF on cell number or [$^3$H] thymidine incorporation into any cell line, showing the action of LMF to be specific for protein synthesis. LMF produced an early (within 30 min) increase in cyclic AMP levels in $C_2C_{12}$ myoblasts, which was linearly ($r^2$ = 0.973, $P$ = 0.004) related to the increase in protein synthesis after 24 h (Figure 3). This suggests that the 2 effects may be related. There was an increase in protein kinase A (PKA) with increasing concentrations of LMF, which reached maximum stimulation at 58 nM LMF and was independent of LMF concentration up to 580 nM ($P$ < 0.05 from control) (Figure 4). The stimulating effect of LMF on protein synthesis in $C_2C_{12}$ myoblasts was attenuated by the adenylate cyclase inhibitor $MDL_{12330A}$, but not by the cyclic AMP-dependent protein kinase inhibitor H8 at 10 μM (Table 1). Both forskolin (25 μM) and dibutryl cyclic AMP (1 μM) stimulated protein synthesis, confirming a role for cyclic AMP in the process. Stimulation of protein synthesis by forskolin, but not by dibutryl cyclic AMP was additive with that of LMF (Table 1). The induction of protein synthesis in $C_2C_{12}$ myoblasts by LMF was partially inhibited by a polyclonal antibody to zinc-$\alpha_2$-glycoprotein (ZAG) (Table 2), and the non-specific β-adrenergic receptor antagonist, propranolol (10 μM) (Figure 5). The specific $\beta_3$-adrenergic receptor antagonist, SR59230A (Nisoli et al, 1996) at a concentration of $10^{-5}$ M significantly reduced LMF stimulation of protein synthesis down to control levels (Figure 6). This suggests that stimulation of protein synthesis by LMF may be mediated through a $\beta_3$-adrenergic receptor. There was no effect on the LMF stimulation of protein synthesis by inhibitors of p70S6 kinase (rapamycin, 0.5 ng ml$^{-1}$), mitogen-activated protein kinase (PD 98059, 0.63 μM), or of phosphatidylinositide-3-OH kinase (wortmannin, 0.02 μM, or LY 294002, 0.05 μM) (Table 2).

Administration of LMF (8 μg, i.v., b.d.) to ex-breeder male NMRI mice caused a progressive decrease in body weight (Figure 7), which became significantly different from control animals administered PBS within 24 h of the first injection. Despite this overall loss of body weight, which was exclusively fat, soleus muscles from LMF treated animals showed a 69% increase in protein synthesis ($P$ = 0.006 from control) (Figure 8A) and a 26% decrease in protein degradation ($P$ = 0.03 from control) (Figure 8B) 48 h after the first injection of LMF. Myosin levels were also increased in soleus muscle from mice receiving LMF (Figure 8C). Densitometric analysis showed a 46 ± 9% ($P$ < 0.05 from control) increase in myosin levels after LMF. In $C_2C_{12}$ myoblasts LMF had no effect on the activity of the lysosomal enzymes, cathepsins L or B (data not shown), but produced a progressive decrease in the functional activity of the proteasome, as determined by the ‘chymotrypsin-like’ enzyme activity, using the fluorogenic substrate succinyl LLVY-MCA (Figure 9A). Western blotting of cellular supernatants of LMF treated cells with MCP231 antibody, a murine monoclonal to the 20S proteasome, which reacts with the α-type subunits, showed a decrease in expression with increase in LMF concentration (Figure 9B) paralleling the decrease in functional activity. Unlike LMF, PIF produced an increase in expression of the 20S proteasome α-subunits, which varied with the concentration, reaching a maximal 77% increase at 10.4 nm PIF (Figure 10A). Addition of LMF (580 nM) completely attenuated the increased expression of the 20S α-subunits in the presence of PIF (Figure 10B).

### DISCUSSION

Loss of skeletal muscle protein is a characteristic feature of cancer cachexia leading to immobility of the patient and eventually to

 *© 2001 Cancer Research Campaign*

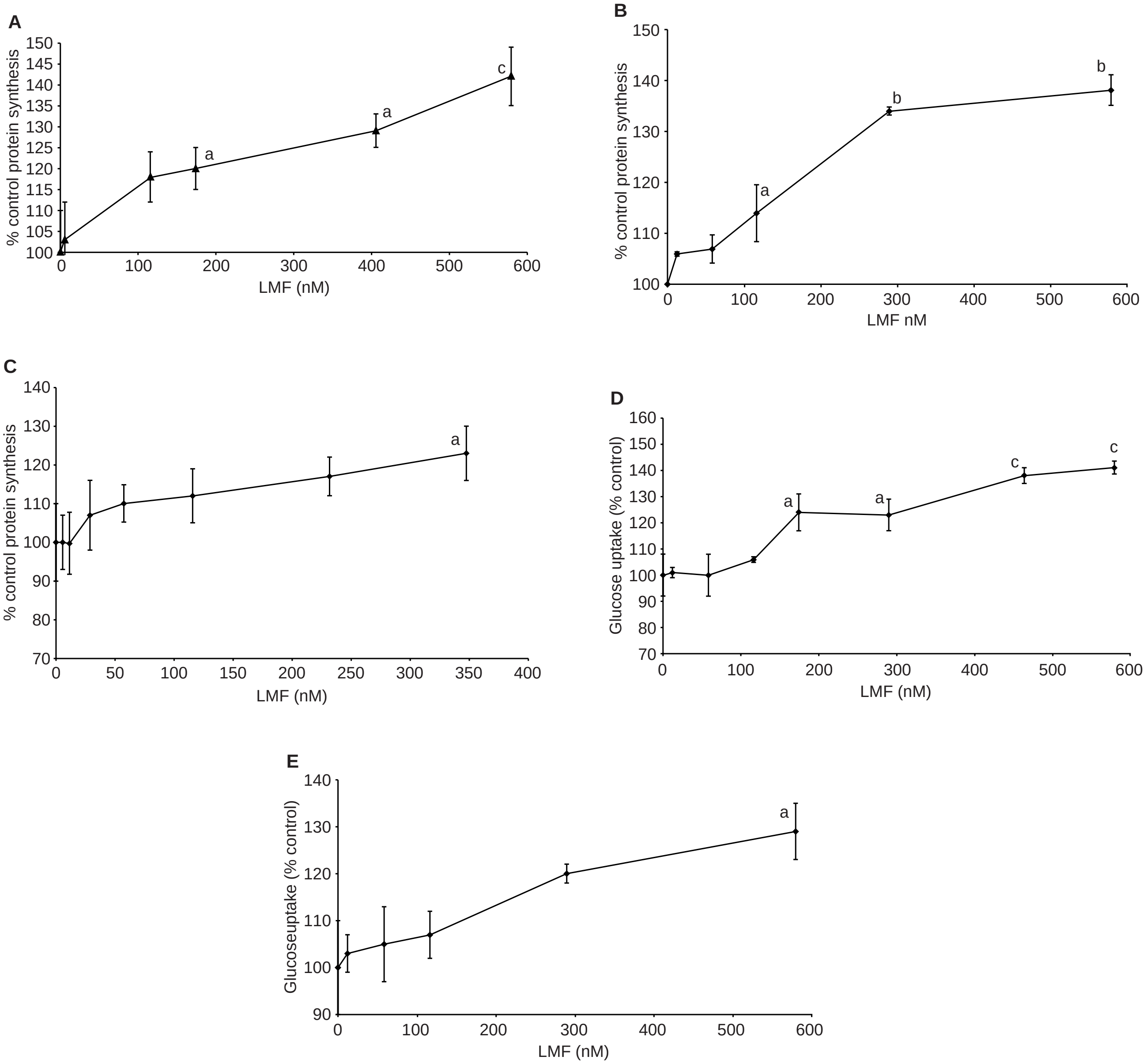

**Figure 2** Dose–response curves showing the effect of LMF on protein synthesis in $C_2C_{12}$ myoblasts (**A**), $C_2C_{12}$ myotubes (**B**) and MAC16 cells (**C**) and 2-deoxyglucose uptake in $C_2C_{12}$ myoblasts (**D**) and MAC16 cells (**E**) after 24 h incubation with LMF. Values are represented as mean ± SEM where $n = 3$ and the experiment was repeated 4 times. Differences from controls incubated in the absence of LMF were determined by one-way ANOVA with Student–Newman–Keuls test and are indicated as a, $P < 0.05$, b, $P < 0.01$ and c, $P < 0.001$

death (Tisdale, 1997). This process has been attributed to tumour production of PIF, which inhibits protein synthesis and increases protein catabolism in skeletal muscle (Todorov et al, 1996; Lorite et al, 1997). However, the present study shows that LMF, also produced by cachexia-inducing tumours, appears to oppose the action of PIF by increasing protein synthesis and decreasing protein degradation in muscle. This action of LMF has been demonstrated in the soleus muscles of mice administered LMF as well as in isolated myoblasts and myotubes. The effect of LMF on protein synthesis was attenuated, both by propranolol, a non-specific β-adrenergic agonist as well as by SR59230A, which has been reported (Nisoli et al, 1996) to have a 10-fold selectivity for the $\beta_3$-over the $\beta_1$-AR, suggesting that the action of LMF may be mediated through a β-AR. β-Adrenergic agonists have been shown to lead to increased muscle protein synthesis, accompanied or followed by decreased protein degradation (Bell et al, 1998), through a cyclic AMP-dependent pathway. The increase in protein synthesis produced by LMF in $C_2C_{12}$ myoblasts was also linearly related to increase in cyclic AMP levels and attenuated by the adenylate cyclase inhibitor, $MDL_{12330A}$. The mechanisms leading from an increase in cyclic AMP to increased protein synthesis have not been fully elucidated.

Gene regulation by polypeptide growth factors is thought to be mediated by transcription factors controlled either by the mitogen-activated protein (MAP) kinase pathway (Davis, 1993) or the p70S6 kinase ($p70^{s6k}$) (Sturgill and Wu, 1991), which rapidly phosphorylates the S6 protein of the 40S ribosomal subunit. Stimulation of protein synthesis in L6 myoblasts was blocked by

*© 2001 Cancer Research Campaign*

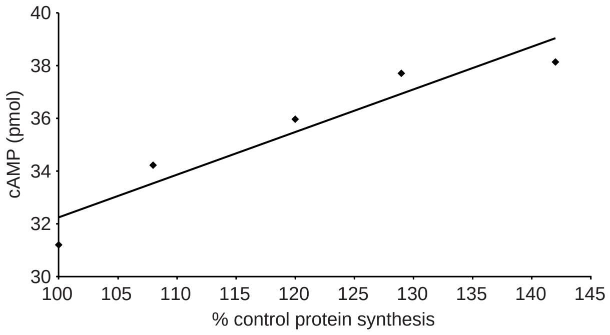

**Figure 3** Relationship between the increase in protein synthesis in $C_2C_{12}$ myoblasts after 24 h incubation with LMF and the intracellular concentration of cyclic AMP, determined after 30 min incubation. $r^2 = 0.973$; $P = 0.0044$

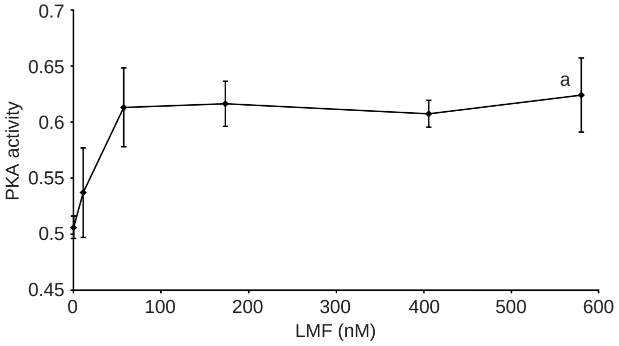

**Figure 4** Effect of LMF on the protein kinase A activity in $C_2C_{12}$ myoblasts determined 24 h after LMF addition. Values are represented as mean ± SEM where $n = 3$ and the experiment was repeated 4 times. Differences from controls incubated in the absence of LMF were determined by one-way ANOVA with Dunnett's test

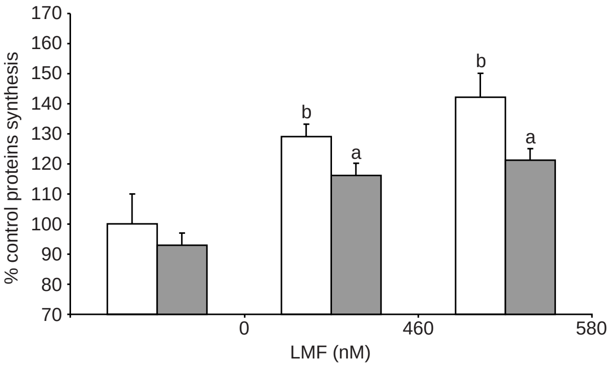

**Figure 5** The effect of the non-specific β-AR antagonist propranolol (10 μM) on LMF-induced protein synthesis in $C_2C_{12}$ myoblasts. Propranolol was added 1 h prior to LMF and protein synthesis was determined 24 h after addition of LMF (closed boxes) and compared with cells treated with LMF alone (open boxes). The values represent means ± SEM where $n = 3$ and the experiment was repeated 4 times. Statistical analysis was performed using one-way ANOVA with Student–Newman–Keuls test and differences are indicated as a, $P < 0.05$ from LMF alone and b, $P < 0.01$ from control

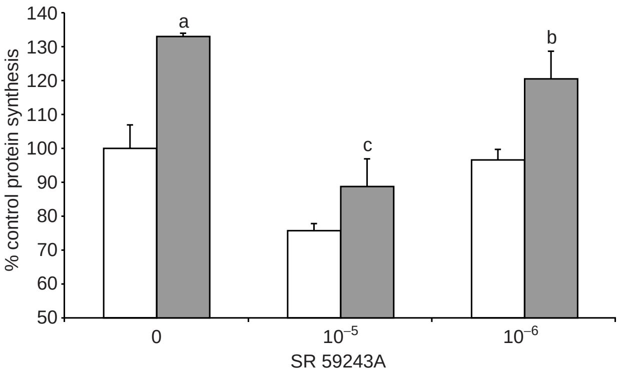

**Figure 6** The effect of the specific β3-AR antagonist. SR59230A on LMF-induced protein synthesis in $C_2C_{12}$ myoblasts. SR59230A was added 1 h prior to the addition of LMF (580 nM) and protein synthesis was determined after a further 24 h in the absence of LMF (open boxes) or in the presence of LMF (closed boxes). The values represent means ± SEM where $n = 3$ and the experiment was repeated 4 times. Statistical analysis was performed using one-way ANOVA with Student–Newman–Keuls test and differences are indicated as a, $P < 0.001$ from cultures not incubated with LMF and b, $P < 0.01$ and c, $P < 0.001$ for protein synthesis in the absence of SR59230A

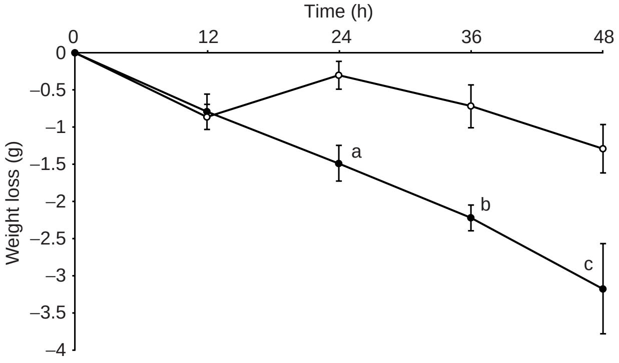

**Figure 7** Effect of i.v. administration of LMF (8 μg, b.d.) on body weight of exbreeder male NMRI mice (●) compared with animals administered PBS (○). Body weight was measured prior to each injection and the weight loss is shown as means ± SEM where $n = 5$. The average body weight of the mice on initiation of the experiment was 44.50 ± 1.13 and was 41.34 ± 0.76 after 48 h treatment with LMF. Differences from control values were determined by Student–Newman–Keuls test and are indicated as a, $P < 0.05$; b, $P < 0.01$ and c, $P < 0.001$.

inhibitors of $p70^{s6k}$ (rapamycin) and MAP kinase (PD-98059), as well as by inhibitors of phosphatidylinositide-3-OH kinase (wortmannin) (Kimball et al, 1998). However, neither rapamycin, PD-98059, wortmannin or the potent and specific phosphatidylinositide-3-OH kinase had any effect on LMF stimulation of protein synthesis in $C_2C_{12}$ murine myoblasts, suggesting an alternative pathway was involved. While in COS-7 cells cyclic AMP activates the MAP kinase pathway (Faure et al, 1994) in rat phaeochromocytoma, PC12, it stimulates the MAP kinase isoenzyme extracellular signal-regulated kinase 1 (ERK1) (Frodin et al, 1994). In addition the ribosomal protein S6 is directly phosphorylated by cyclic AMP-dependent protein kinase (Wettenhall and Cohen, 1992). Transcriptional regulation following stimulation of adenylate cyclase can also be mediated by the family of cyclic AMP-response element (CRE)-binding proteins (Habener, 1990). These factors are phosphorylated by PKA with increasing concentrations of cyclic AMP, leading to high stimulation in the transactivating potential (de Groot et al, 1993). These CRE-binding proteins may be involved in LMF stimulation of protein synthesis, although the

 © 2001 Cancer Research Campaign

**Table 1** The effect of agents influencing intracellular cyclic AMP on LMF-induced protein synthesis in $C_2C_{12}$ myoblasts[a]

| Treatment | % Control protein synthesis (± SEM) | *P* value[b] | |
|---|---|---|---|
| | | From control | From LMF |
| LMF (580 nM) | 136 ± 7 | <0.001 | – |
| $MDL_{12330A}$ (20 µM) | 96 ± 10 | NS | – |
| $MDL_{12330A}$ (20 µM) + LMF | 113 ± 14 | – | < 0.05 |
| H8 (10 µM) | 104 ± 3 | NS | – |
| H8 (10 µM) + LMF | 125 ± 2 | – | NS |
| Forskolin (25 µM) | 158 ± 7 | <0.001 | – |
| Forskolin (25 µM) + LMF | 178 ± 5 | – | < 0.001 |
| Dibutyryl cyclic AMP (1 µM) | 125 ± 4 | <0.01 | – |
| Dibutyryl cyclic AMP (1 µM) + LMF | 148 ± 3 | – | NS |

[a]Agents were added 1 h prior to LMF and protein synthesis determined after 24 h. [b]Statistical analysis performed using one-way ANOVA with Student–Newman–Keuls test.

**Table 2** The effect of potential inhibitors on LMF-induced protein synthesis in $C_2C_{12}$ myoblasts[a]

| Treatment | % Control protein synthesis (± SEM) | *P* value[b] | |
|---|---|---|---|
| | | From control | From LMF |
| LMF (406 nM) | 132 ± 8 | < 0.01 | – |
| LY294002 (50 nM) | 89 ± 3 | NS | – |
| LY294002 (50 nM) + LMF | 128 ± 10 | – | NS |
| Wortmannin (20 nM) | 77 ± 10 | < 0.05 | – |
| Wortmannin (20 nM) + LMF | 107 ± 6 | – | NS[c] |
| Rapamycin (0.5 ng $ml^{-1}$) | 62 ± 7 | < 0.001 | – |
| Rapamycin (0.5 ng $ml^{-1}$) + LMF | 93 ± 2 | – | NS[c] |
| PD98059 (625 nM) | 92 ± 5 | NS | – |
| PD98059 (625 nM) + LMF | 129 ± 4 | – | NS |
| Anti-ZAG (10 µg $ml^{-1}$) | 100 ± 5 | NS | – |
| LMF (406 nM) + Anti-ZAG | 120 ± 7 | – | < 0.05 |
| LMF (580 nM) | 142 ± 6 | < 0.01 | – |
| LMF (580 nM) + Anti-ZAG | 120 ± 6 | – | < 0.05 |

[a]Agents were added 1 h prior to LMF and protein synthesis determined after 24 h. [b]Statistical analysis performed using one-way ANOVA with Student–Newman–Keuls test. [c]Based on new reduced control value.

lack of inhibition of PKA by H8 at 10 µM would negate against the possibility. However, it is possible that higher concentrations of H8 are required for inhibition of PKA in this system. This requires further investigation.

Despite the stimulatory effect of LMF on protein synthesis there was no effect on DNA synthesis or cell number, suggesting a hypertrophic response to this tumour factor. Nevertheless protein synthesis was also enhanced in tumour cells suggesting that LMF is potentially a growth factor for the tumour. LMF also stimulated 2-deoxyglucose uptake into $C_2C_{12}$ myoblasts which suggests that it facilitates glucose utilization. Administration of LMF to mice produces a decrease in blood glucose (Hirai et al, 1998) confirming the ability to stimulate glucose utilization. Li and Adrian (1999) have also reported that pancreatic cancer cells produce a bioactive factor which stimulates glucose uptake and utilization in murine myoblasts. Although the reported $M_r$ of this factor is much lower than that of LMF, in separate experiments (unpublished) we have shown LMF to undergo tryptic cleavage to yield a bioactive fragment of comparable molecular weight.

In addition to stimulation of protein synthesis LMF also attenuates protein catabolism in skeletal muscle. The ubiquitin–proteasome system is considered to be the major pathway for selective protein breakdown in muscle, while lysosomal proteolysis plays only a minor role (Attaix and Taillander, 1998). While there is no effect of LMF on the lysosomal proteolytic enzymes cathepsins B and L, there is a significant inhibition of proteasome catalytic activity, through a decreased expression of the proteasome α-type subunits. Since protein catabolism in cachexia appears to be due to an up-regulation of proteasome expression (Lorite et al, 1998) then LMF appears to be antagonistic to tumour proteolytic factors, such as PIF, and as such may modulate the rate of loss of skeletal muscle mass. In the MAC16 murine cachexia model LMF bioactivity is maximally elevated at 15% weight loss and thereafter declines (Groundwater et al, 1990). In this model a decrease in protein synthesis and an increase in protein degradation is not seen until the weight loss exceeds 16% (Smith and Tisdale, 1993). This suggests that either PIF production is not apparent at low weight loss or that LMF attenuates the action of PIF. This can be determined by administration of the pure factors to mice.

Thus LMF increases muscle mass by an increase in protein synthesis and a decrease in protein catabolism. The effect on protein synthesis appears to arise from increases in intracellular cyclic AMP, possibly mediated through stimulation of a $\beta_3$-adrenergic receptor. Since LMF was also found to stimulate

© 2001 Cancer Research Campaign 

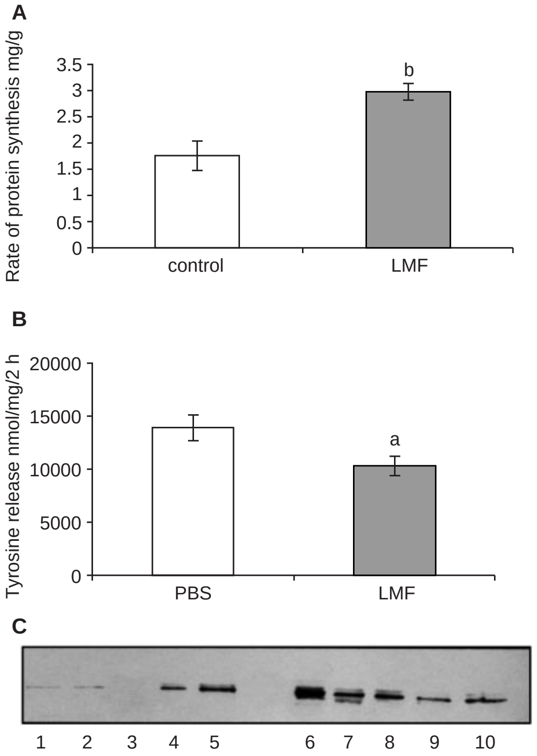

**Figure 8** Effect of LMF on protein synthesis (**A**) and protein degradation (**B**) in soleus muscle of exbreeder male NMRI mice in comparison with animals receiving PBS. Values are shown as means ± SEM where $n = 6$. Differences from control values were determined by Student's *t*-test and are indicated as a, $P = 0.03$ and b, $P = 0.006$. (**C**) Western blot analysis of soluble extracts of gastrocnemius muscle of mice administered PBS (lanes 1–5) or LMF (lanes 6–10) detected with a mouse monoclonal antibody to myosin. Blots were developed by ECL

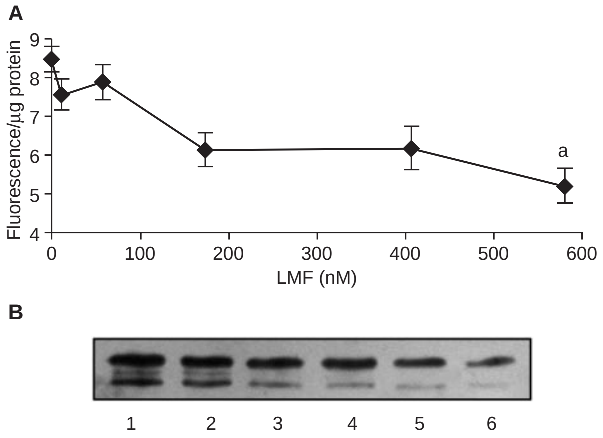

**Figure 9** (**A**) Chymotryptic activity of soluble extracts of $C_2C_{12}$ myoblasts 24 h after treatment with LMF determined using the fluorogenic substrate suc LLVY-MCA. Results are shown as mean ± SEM where $n = 3$ and the experiment was repeated 4 times. Differences from controls in the absence of LMF are indicated as a, $P < 0.05$. (**B**) Western blot analysis of soluble extracts of $C_2C_{12}$ myoblasts from cells treated with PBS (lane 1) or 11.6 nM (lane 2); 58 nM (lane 3); 174 nM (lane 4); 406 nM (lane 5) or 580 nM (lane 6) LMF for 24 h and detected with a mouse monoclonal antibody to the 20S proteasome α-subunits. The blot was developed by ECL. Densitometric analysis showed LMF (nM) to produce the following percentage of the control (lane 1), 11.6 nM (89%); 58 nM (76%); 174 nM (52%); 406 nM (47%); 580 nM (35%). A parallel gel was silver-stained to ensure equal loading

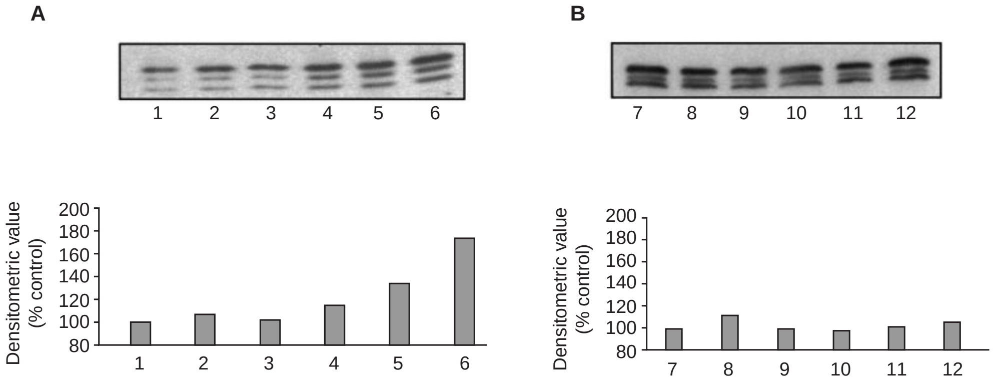

**Figure 10** (**A**) Western blot analysis and densitometric scans of soluble extracts of $C_2C_{12}$ myoblasts treated with PBS (lane 1) or 1 nM (lane 2); 2 nM (lane 3); 5 nM (lane 4); 8.3 nM (lane 5); 10 nM (lane 6) PIF for 24 h and detected with a mouse monoclonal antibody to the 20S proteasome α-subunits. (**B**) Western blot analysis of $C_2C_{12}$ myoblasts treated with increasing concentrations of PIF for 24 h in the presence of LMF (580 nM). The lanes (7–12) represent the same concentrations of PIF as depicted in (**A**)

*© 2001 Cancer Research Campaign*

protein synthesis in tumour cells it may function to increase overall tumour bulk without affecting cellular proliferation.

### ACKNOWLEDGEMENTS

We gratefully acknowledge Professor KC Fearon and his team at Department of Surgery, Edinburgh Royal Infirmary, Scotland for the provision of the cancer patient urine.

### REFERENCES

Anderson PT, Helferich WG, Parkhill LC, Merkel RA and Bergen WG (1990) Ractopamine increases total and myofibrillar protein synthesis in cultured rat myotubes. *J Nutr* **120**: 1677–1683

Attaix P and Taillander D (1998) The critical role of the ubiquitin-proteasome pathway in muscle wasting in comparison to lysosomal and $Ca^{2+}$-dependent systems. *Adv Mol Cell Biol* **27**: 235–266

Bell AW, Bauman DE, Beerman DH and Harrell RJ (1998) Nutrition, development and efficacy of growth modifiers in livestock species. *J Nutr* **128**: 360S–363S

Davis RJ (1993) The mitogen-activated protein kinase signal transduction pathway. *J Biol Chem* **268**: 14553–14556

de Groot RP, den Hertog J, Vandenheede JR, Goris J and Sassome-Corsi R (1993) Multiple and cooperative phosphorylation events regulate the CREM activator function. *EMBO J* **12**: 3903–3911

Faure M, Yoyno-Yasenetskaya TA and Bourne HR (1994) cAMP and βγ subunits of heterotrimeric G proteins stimulate the mitogen-activated protein kinase pathway in COS-7 cells. *J Biol Chem* **269**: 7851–7859

Frodin M, Peraldi P and Van Obberghen E (1994) Cyclic AMP activates the mitogen-activated protein kinase cascade in PC12 cells. *J Biol Chem* **269**: 6207–6214

Grant AL, Helferich WG, Merkell RA and Bergen WG (1990) Effects of phenethanolamine and propranolol on the proliferation of cultured chick breast muscle satellite cells. *J Anim Sci* **68**: 652–658

Groundwater P, Beck SA, Barton C, Adamson C, Ferrier IN and Tisdale MJ (1990) Alteration of serum and urinary lipolytic activity with weight loss in cachectic cancer patients. *Br J Cancer* **62**: 816–821

Habener JF (1990) Cyclic AMP-response element binding proteins: a cornucopia of transcription factors. *Mol Endocrinol* **4**: 1087–1094

Hirai K, Hussey HJ, Barber MD, Price SA and Tisdale MJ (1998) Biological evaluation of a lipid-mobilizing factor isolated from the urine of cancer patients. *Cancer Res* **58**: 2359–2365

Khan S and Tisdale MJ (1999) Catabolism of adipose tissue by a tumour-produced lipid-mobilising factor. *Int J Cancer* **80**: 444–447

Kimball SR, Horetsky RL and Jefferson LS (1998) Signal transduction pathways involved in the regulation of protein synthesis by insulin in L6 myoblasts. *Am J Physiol* **274**: C221–C228

Li J and Adrian TE (1999) A factor from pancreatic and colon cancer cells stimulates glucose uptake and lactate production in myoblasts. *Biochem Biophys Res Commun* **260**: 626–633

Lorite MJ, Cariuk P and Tisdale MJ (1997) Induction of muscle protein degradation by a tumour factor. *Br J Cancer* **76**: 1035–1040

Lorite MJ, Thompson MG, Drake JL, Carling G and Tisdale MJ (1998) Mechanism of muscle protein degradation induced by a cancer cachectic factor. *Br J Cancer* **78**: 850–856

Mersmann HJ (1998) Overview of the effects of β-adrenergic receptor agonists on animal growth including mechanisms of action. *J Anim Sci* **76**: 160–172

McMillan DN, Noble BS and Maltin CA (1992) The effect of the β-adrenergic agonist clenbuterol on growth and protein metabolism in rat muscle cell cultures. *J Anim Sci* **70**: 3014–3023

Nisoli E, Tonello C, Landi M and Carruba MO (1996) Functional studies of the first selective β3-adrenergic receptor antagonist SR59230A in rat brown adipocytes. *Mol Pharm* **49**: 7–14

Orino E, Tanaka K, Tamura T, Sone S, Ogura T and Ichihara A (1991) ATP-dependent reversible association of proteasomes with multiple protein components to form 26S complexes that degrade ubiquitinated proteins in human HL-60 cells. *FEBS Lett* **284**: 206–210

Smith KL and Tisdale MJ (1993) Increased protein degradation and decreased protein synthesis in skeletal muscle during cancer cachexia. *Br J Cancer* **67**: 680–685

Southorn BG and Palmer RM (1990) Inhibitors of phospholipase $A_2$ block the stimulation of protein synthesis by insulin in L6 myoblasts. *Biochem J* **270**: 737–739

Stefanovic D, Erickson E, Pike LJ and Maller JL (1986) Activation of a ribosomal protein S6 protein kinase in xenopus oocytes by insulin and insulin-receptor kinase. *EMBO J* **5**: 157–160

Sturgill TW and Wu J (1991) Recent progress in characterization of protein kinase cascades for phosphorylation of ribosomal protein S6. *Biochim Biophys Acta* **1092**: 350–357

Tisdale MJ (1997) Biology of cachexia. *J Natl Cancer Inst* **89**: 1763–1773

Todorov P, Cariuk P, McDevitt T, Coles B, Fearon K and Tisdale M (1996) Characterization of a cancer cachectic factor. *Nature* **379**: 739–742

Waalkes TP and Udenfriend SA (1957) A fluorometric method for the estimation of tyrosine in plasma and tissues. *J Lab Clin Med* **50**: 733–736

Wettenhall REH and Cohen P (1982) Isolation and characterisation of cyclic AMP-dependent phosphorylation sites from rat liver ribosomal protein S6. *FEBS Lett* **140**: 263–269

© 2001 Cancer Research Campaign
